# Retraction: A Novel Image Recuperation Approach for Diagnosing and Ranking Retinopathy Disease Level Using Diabetic Fundus Image

**DOI:** 10.1371/journal.pone.0181891

**Published:** 2017-07-18

**Authors:** The PLOS ONE Editors

It has been brought to the attention of the *PLOS ONE* editors that this article contains substantial overlap in text and scientific content with the publication below by the same authors:

Diagnosing and ranking retinopathy disease level using diabetic fundus image recuperation approach. ScientificWorldJournal. 2015;2015:534045. doi: 10.1155/2015/534045.

Figures 1 and 2 duplicate images included in the publication in *The Scientific World Journal*. There is also substantial overlap in the Introduction, Methods, Discussion and Conclusion sections.

The *PLOS ONE* editors retract this article as we consider that this constitutes redundant publication.
